# Effect of Selective Laser Melting Process Parameters on the Quality of Al Alloy Parts: Powder Characterization, Density, Surface Roughness, and Dimensional Accuracy

**DOI:** 10.3390/ma11122343

**Published:** 2018-11-22

**Authors:** Ahmed H. Maamoun, Yi F. Xue, Mohamed A. Elbestawi, Stephen C. Veldhuis

**Affiliations:** Department of Mechanical Engineering, McMaster University, 1280 Main Street, West Hamilton, ON L8S 4L7, Canada; xueyf4@mcmaster.ca (Y.F.X.); veldhu@mcmaster.ca (S.C.V.)

**Keywords:** additive manufacturing, selective laser melting, AlSi10Mg, Al6061, SLM process parameters, performance characteristics

## Abstract

Additive manufacturing (AM) of high-strength Al alloys promises to enhance the performance of critical components related to various aerospace and automotive applications. The key advantage of AM is its ability to generate lightweight, robust, and complex shapes. However, the characteristics of the as-built parts may represent an obstacle to the satisfaction of the parts’ quality requirements. The current study investigates the influence of selective laser melting (SLM) process parameters on the quality of parts fabricated from different Al alloys. A design of experiment (DOE) was used to analyze relative density, porosity, surface roughness, and dimensional accuracy according to the interaction effect between the SLM process parameters. The results show a range of energy densities and SLM process parameters for AlSi10Mg and Al6061 alloys needed to achieve “optimum” values for each performance characteristic. A process map was developed for each material by combining the optimized range of SLM process parameters for each characteristic to ensure good quality of the as-built parts. This study is also aimed at reducing the amount of post-processing needed according to the optimal processing window detected.

## 1. Introduction

High-strength aluminum alloys (Al alloys) are typically used for the production of lightweight critical components for a variety of applications in space, aerospace, automotive, military, and biomedical fields [1]. Additive manufacturing (AM) offers additional flexibility in the design and manufacturing of parts, particularly the ability to fabricate complex geometries without the need for custom tools [2]. Selective laser melting (SLM) offers superior dimensional accuracy and material quality of the fabricated parts [3].

SLM is a layer-by-layer process, in which the laser beam selectively melts the powder layer according to slices generated from the three-dimensional designed model. SLM possesses rapid melting and solidification rates and thus is applicable for a narrow selection of materials according to their coefficient of thermal expansion (CTE). In addition, the optimization of the SLM process parameters of Al alloys is hampered by part defects due to energy loss in the laser beam projected to the powder bed surface. The quality of Al alloys produced by SLM could be influenced by the chemical composition and CTE of the material used. Galy et al. [4] showed that porosity, hot cracking, anisotropy, and surface quality are the principal defects of Al alloy parts. They also demonstrated that the selection of SLM process parameters and the laser beam energy loss due to Al reflectivity are the primary causes of porosity and hot crack formation. The pre-mixing of Al alloy powder with some composite materials might improve the material properties of the SLM as-built parts [5,6]. However, the quality and material properties of Al alloy parts can be customized according to the SLM parameters selected without pre-mixing with other elements.

Some of the SLM process parameters can be controlled, such as laser power, scan speed, hatch spacing, and powder layer thickness. The energy density is a function of these parameters. Optimization of the SLM process parameters is an essential step for controlling material characteristics and the quality of the fabricated parts. Sufiiarov et al. [7] showed that a 30 µm powder layer thickness could result in higher strength and lower elongation for Inconel 718 than a 50 µm layer thickness. Nguyen et al. [8] also studied the effect of the powder layer thickness within a range from 20 to 50 µm. Their results showed that, as the thickness of the powder layer diminishes, part density and dimensional accuracy increase. Cheng et al. [9] investigated the effect of the scanning strategy on the stress and deformation of parts. Their results showed that minimum stress and deformation values are obtained using a layer orientation strategy with an angle of 45° or 67°. The powder feedstock quality also represents an essential parameter that might affect part characteristics. Sutton et al. [10] reported that the powder morphology, microstructure, and chemical characteristics could change depending on the production method, i.e., gas, water, or plasma atomization. This could generate a difference in quality between the parts produced using different feedstock powders [11,12].

Various studies [13,14,15,16,17] utilized a design of experiment (DOE) approach to investigate the effect of SLM process parameters on AlSi10Mg part quality, by evaluating their density, surface roughness, and dimensional accuracy. Read et al. [14] used the response surface methodology (RSM) to evaluate the influence of SLM process parameters on part porosity. Their study was limited by the use of a laser power up to 200 W. The results showed that minimum porosity was obtained at a critical energy density of 60 J/mm^3^. Abouelkhair et al. [15] used the one-factor-at a-time (OFAT) method to optimize the SLM process parameters for producing dense parts. They achieved an optimum combination of laser power, scan speed, and hatch spacing, which resulted in a 99.77% relative density. Hitzler et al. [17] demonstrated that the surface roughness of the as-built samples varies according to their position on the build plate. They also concluded that the increase of energy density resulted in higher values of roughness on the surface of the side faces, compared to the roughness values measured on the top surface. Calignano et al. [16] used the Taguchi method to investigate the effect of the SLM process parameters on the surface roughness of the parts. They found that the laser scan speed has a significant influence on the surface roughness. Lower surface roughness was obtained using a scan speed of 900 mm/s, 120 W laser power, and 0.1 mm hatch spacing. Han et al. [18] reported that a decrease in surface roughness, combined with an increase of the laser scan speed, results in better dimensional accuracy. It is worthwhile to note that the previous studies used the DOE within a range of laser power up to 200 W. However, the post-processing treatment is also considered to be an essential stage for reducing the defects inside the as-built parts. This, in turn, raises the final production cost of the parts [19,20,21]. Consequently, the optimization of the SLM process parameters has a significant role in optimizing the steps of the manufacturing process. This might lead to a cost-effective process for specific applications which are compatible with the characteristics of the as-built parts. 

In general, Al6061 is seldom used for SLM. Fulcher et al. [22] reported that Al6061 parts have a lower dimensional accuracy compared to AlSi10Mg parts because of a higher CTE. High-strength Al alloys such the Al6061 and Al7075 series have low Mg and Si content, which might result in hot cracking and formation of large columnar grains [23]. Louvis et al. [24] reported that low-relative-density parts of Al6061 might be produced via SLM because of the effect of oxide formation inside these parts. This might result from the relatively low laser power used, (100 W) which may not be enough to achieve complete melting. In general, more research is required to evaluate the effect of SLM process parameters on the as-built Al6061 characteristics such as density, surface roughness, and dimensional accuracy. In addition, the effect of Si content requires further investigation aimed at optimizing the process parameters.

In this study, a comprehensive experimental work using the DOE approach was performed to evaluate the influence of the SLM process parameters on the quality of as-built Al alloys. The current work focuses on investigating the density, surface topology, and dimensional accuracy of AlSi10Mg and Al6061. SLM process parameters were selected over a wide range of laser power, scanning speed, and hatch spacing values. Part characteristics were evaluated for various SLM parameters to develop a process map which displayed the effect of Si content on part quality. Maamoun et al. [25] also covered the impact of the SLM process parameters on the microstructure and mechanical properties of the same Al alloys. This work aims to investigate the limits of SLM in fabricating critical components for the aerospace industry using these alloys. In particular, the current research is focused on producing high-quality metallic optics and optomechanical components to improve the performance of telescopes and laser systems. 

## 2. Experimental Procedure

### 2.1. Material

Powder characterization was performed according to ASTM F3049-14. The powders’ chemical composition was evaluated using Energy X-ray dispersive Spectroscopy (EDS) equipped in TESCAN VP Scanning Electron Microscope (SEM). The powder size distribution (PSD) was measured using laser diffraction by dispersing the powder in water. PSD is attributed by D (α), which represents the diameter of the measured particle, where α is the particles volume percentage that have a smaller diameter than the D value. The powder morphology was investigated using the SEM instrument. A Bruker D8 DISCOVER diffractometer equipped with a cobalt sealed tube source and an area detector was used to obtain the X-ray diffraction (XRD) phase pattern for both powders.

### 2.2. Design of Experiment

A DOE was developed to evaluate the response of the SLM process parameters and the volumetric energy density with respect to the as-built parts’ quality. The volumetric energy density is defined as follows:

(1) $$E_{d}=\frac{P}{V_{s}*D_{h}*T_{l}}$$

where E_d_ is the energy density (J/mm^3^), P is the laser beam power (W), V_s_ is the laser scan speed (mm/s), D_h_ is the hatch spacing between scan passes, and T_l_ is the deposited layer thickness (µm). The OFAT method was used to analyze the performance of AlSi10Mg samples. Eight different samples were produced with six replications for each. Several SLM parameters were selected to build the AlSi10Mg samples, as listed in Table 1, with a constant layer thickness of 30 µm. The effect of the laser power, scan speed, hatch spacing, and energy density on the as-built part characteristics were evaluated with regression analysis. 

A full factorial DOE was developed using the response surface over a wide range of SLM parameters. Two sets of three SLM parameters (laser power, scanning speed, and hatch spacing) were selected, as presented in Table 2. Three samples for each SLM parameters group were fabricated for a total of 48 samples. The energy density (E_d_) for the Al6061 study was selected within a higher range (40–125 J/mm^3^) compared to the E_d_ used for AlSi10Mg (27–65 J/mm^3^). This was due to the higher reflectivity of the laser power for Al6061, which resulted in less energy absorption by the powder particles. The overlap of SLM parameters for some samples of AlSi10Mg and Al6061 enabled the investigation of each material at equal parameters. The correlation coefficient (R^2^) was used to indicate how the regression models fit with the measured data, and this factor was added to each performance characteristic map for each material. 

### 2.3. SLM Process Parameters

The AlSi10Mg and Al6061 parts were fabricated by an EOSINT M290 machine equipped with a 400 W Yb-fiber laser using a 100 µm laser beam diameter. The same layer thickness of 30 µm and layer orientation angle of 67° were selected for all samples undergoing strip scan, using a 0.02 mm laser beam offset. The build chamber was vacuumed with argon to reduce the oxygen content below 0.1%, and thus the possibility of oxide formation in the produced parts. All samples were fabricated as 15 mm cubes according to the SLM parameters listed in Table 1 and Table 2. A preheating technique was applied to the build platform at 200 °C before starting the build, to minimize the thermal residual stresses (by reducing the thermal gradient between the deposited layers). 

### 2.4. Sample Characterization Method

In the current study, the as-built part characterization focuses on relative density, internal porosity, surface roughness, and dimensional accuracy. Archimedes method was used to measure the density of the as-built cubes for both AlSi10Mg and Al6061 samples. The relative density was also evaluated after sample surface polishing to investigate the percentage of internal porosity. Density measurement via water displacement, according to ASTM B962-17, was performed with an electronic densimeter (MD-200S).

Surface roughness measurements were performed according to ASTM D7127-17 with a Mitutoyo SJ-410 surface tester. Five measurements at intervals of 4.5 mm were conducted on the cubic specimen’s top surface, and their average was taken at each location. A light microscope (Alicona Infinite Focus G5) was used to capture the surface texture of some of the AlSi10Mg and Al6061 samples. The area tested was 10 mm × 10 mm using a 10× magnification lens, and surface roughness was also measured to validate the values obtained by the mechanical stylus.

The measurement of geometric dimensions and tolerances (GD&T) was conducted with a Mitutoyo CRYSTA-Apex S544 Coordinate Measuring Machine (CMM) which includes an SP25M stylus. This machine has a resolution of 0.1 µm within a working zone of 500 mm × 400 mm × 400 mm. The tested surface was probed at 10 measurement points along each sample’s face. Flatness, perpendicularity, and parallelism were measured for all sample faces, except for the bottom.

## 3. Results and Discussion

### 3.1. Powder Characterization

**Figure 1:**
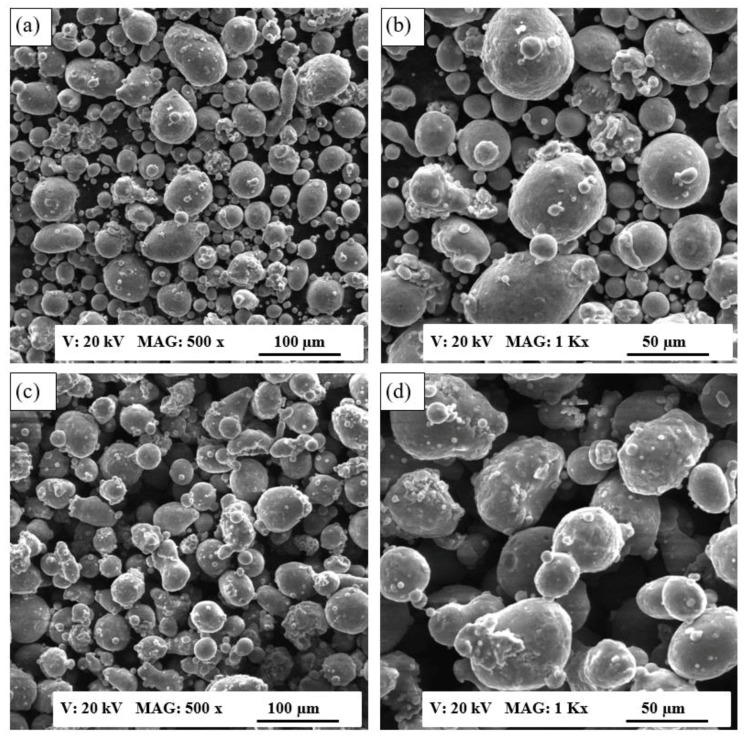
SEM observations of the powder morphology: (**a**,**b**) Al6061 powder, (**c**,**d**) AlSi10Mg powder.

The characteristics of the gas-atomized AlSi10Mg and Al6061 powders, supplied by the LPW Technology, Imperial, USA, were examined according to ASTM F3049-14. The powders were sieved with a 75 µm mesh before being characterized. The morphology of both powders was detected using SEM, as illustrated in Figure 1. The SEM observations showed a relatively higher percentage of elongated or irregular-shape particles in the AlSi10Mg powder compared to the same alloy provided by a different supplier, which presented spherical particles, as reported by Maamoun et al. [19,20]. Figure 1a,b shows that the Al6061 powder also had a greater percentage of spherical particles compared to the AlSi10Mg powder shown in Figure 1c,d. The existence of irregular or elongated particles might reduce powder flowability and the homogeneity of the powder layer distribution and thus negatively affect the quality and density of the fabricated parts [11]. The combination of a wide range of fine and coarse particles could increase the powder packing density but it reduces the flowability as a result of the effect of powder cohesion and inter-particle forces [11]. The weight percentages of the chemical elements of both powders were detected with EDS, as listed in Table 3. The results revealed higher Si content and relatively lower weight percentages of Mg, Cu, and Fe inside the AlSi10Mg powder compared to the Al6061 powder. The influence of this difference in chemical composition, in addition to the effect of the powders’ particle shape, on the characteristics of the as-built parts will be discussed in the following sections.

**Figure 2:**
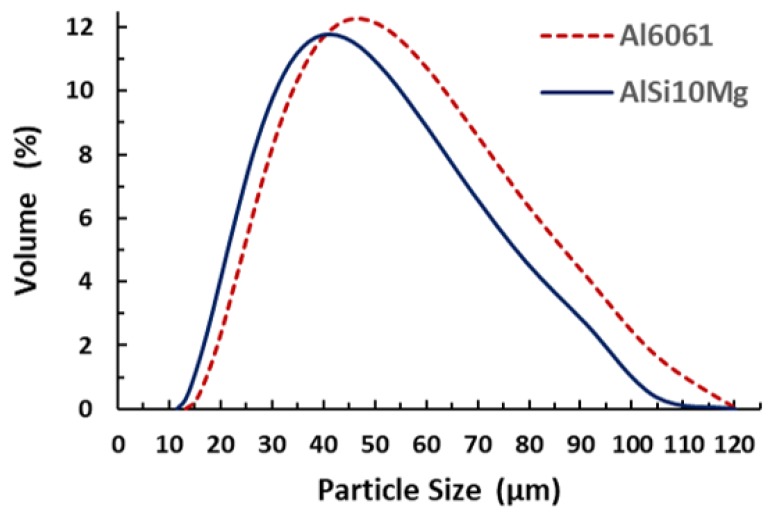
Particle size distribution of the Al6061 and AlSi10Mg powders.

Figure 2 illustrates the particle size distribution (PSD) profile of the AlSi10Mg and Al6061 powders, showing a positively skewed profile. This PSD profile could achieve a better surface quality and higher density compared to negatively skewed and Gaussian distribution grades, by increasing the laser energy absorption [11,26]. The quantitative data of PSD presented in Table 4 show that the particle size ranged from 12 to 110 µm for AlSi10Mg and from 12 to 120 µm for the Al6061 powder. These results indicated the presence of larger sized particles compared to the mesh size used for sieving. This might be related to the detected elongated particles with a smaller cross section which permits filtration through the mesh during sieving. Table 4 data also illustrate that in the AlSi10Mg and Al6061 powders, 90% of the particles were smaller than the sieving mesh (75 µm), with D (0.9) corresponding to 66.55 and 71.92 µm, respectively.

**Figure 3:**
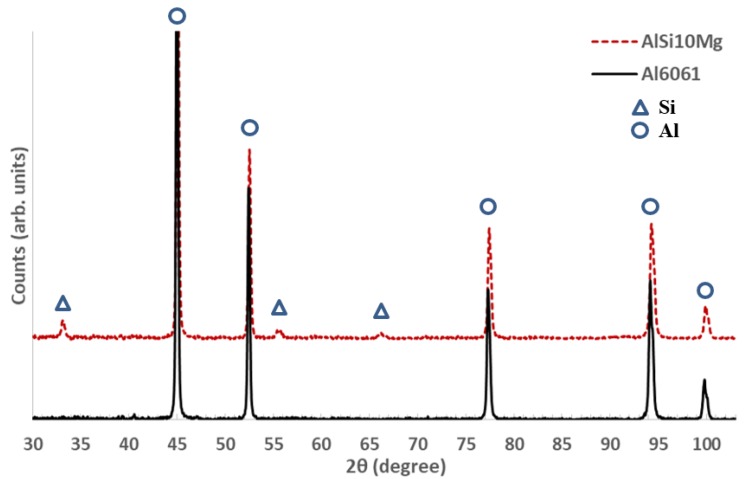
XRD phase patterns of the Al6061 and AlSi10Mg powders.

The XRD phase patterns of the AlSi10Mg and Al6061 powders were detected as shown in Figure 3. The Al and Si peaks were identified according to the Joint Committee on Powder Diffraction Standards (JCPDS) patterns of 01-089-2837 and 01-089-5012, respectively. The low-intensity of the Si peaks in the Al6061 phase pattern was due to its small weight percentage inside that alloy. For the AlSi10Mg powder, the higher intensity of the Si peaks and the slight shift of the Al peaks to the left indicated a lower solubility of Si in AlSi10Mg compared to Al6061 [27].

### 3.2. Relative Density

**Figure 4:**
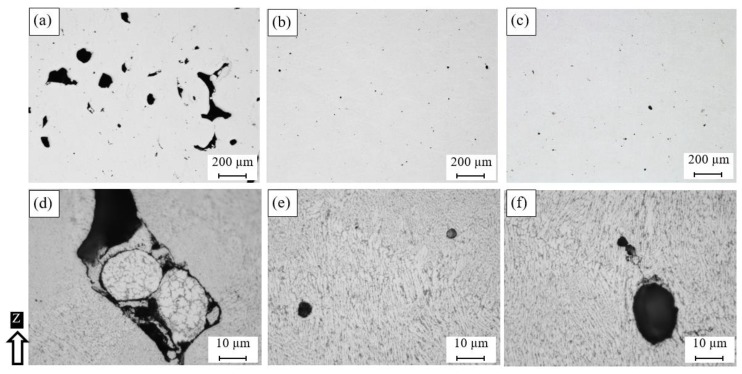
Pores observed inside the as-built AlSi10Mg sample fabricated with different SLM parameters; (**a**,**d**) AS8, (**b**,**e**) AS3, and (**c**,**f**) AS1.

Figure 4 shows the effect of energy density on the formation of pores inside the as-built AlSi10Mg part. The results showed that the low energy density of 27 J/mm^3^ in the AS8 sample could cause significant keyhole porosity due to the lack of fusion of the powder particles during the SLM process, as shown in Figure 4a. The keyhole pores were observed along the building direction within the layer boundaries, which had an irregular elongated shape due to the low energy density. The keyhole pores might form either because of insufficient energy delivered to the powder particles or because of the entrapment of gas bubbles between the interlayers during laser scanning [28]. The keyhole pore size reached 200 µm and gradually decreased as the energy density increased, until disappearing when the energy density value exceeded 50 J/mm^3^. Inside the keyhole pores, partially melted particles were visible, as shown in Figure 4d. This might have occurred owing to the trapping of the consolidated powder inside the keyhole pores as a result of the low energy incident on the powder particle surface. However, spherical hydrogen pores or metallurgical pores with a size within 10 µm were observed at 50 J/mm^3^, as shown in Figure 4b,e. The average size of these spherical pores tended to grow to more than 20 µm at 65 J/mm^3^, as illustrated in Figure 4c,f. The mechanism of pore formation at high fusion rates might be related to the pores existing inside the gas-atomized powder particles [4]. They may also result from the balling phenomena where the melted powder fails to wet the previously deposited layer [29]. The melt pool viscosity might also change according to the applied energy density, and this could affect the porosity formation inside the as-built parts [30].

**Figure 5:**
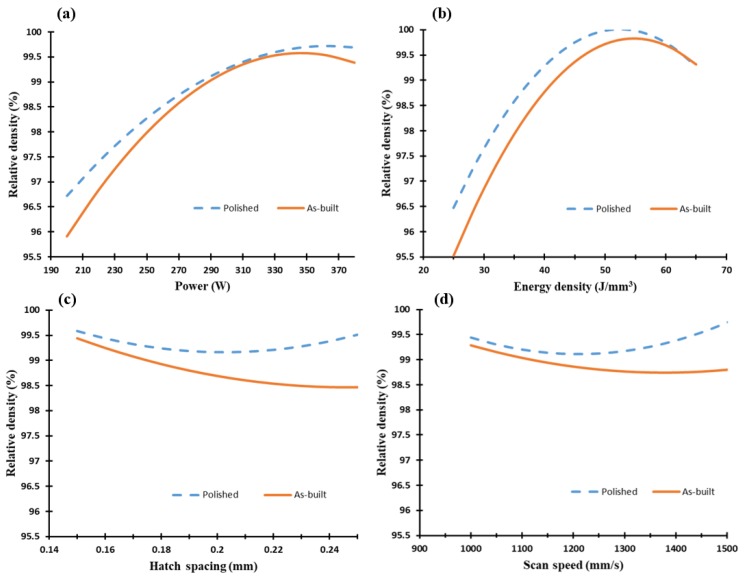
Relative density of the as-built AlSi10Mg samples vs. (**a**) laser power (W), (**b**) energy density (J/mm^3^), (**c**) hatch spacing (mm), and (**d**) scan speed (mm/s).

The mechanism of pore formation inside the as-built AlSi10Mg samples, according to the SLM process parameters presented in Figure 4, was validated after evaluating the relative density. Figure 5 illustrates the map developed by the regression model generated from the DOE analysis. This map describes the effect of laser power, scan speed, hatch spacing, and energy density on the relative density of the as-built AlSi10Mg parts. The results showed the optimum range of the process parameters which allowed the least amount of spherical and keyhole pores to reach the highest possible relative density value of the part. An energy density value between 50 and 60 J/mm^3^ produced a high relative density reaching 99.7%. Beyond this range, the relative density diminished because of the lack of fusion at the lower energy density, balling formation at the higher energy density, or hydrogen gases trapped inside the powder particles. It is worthwhile to note that higher values of the as-built part density could be obtained according to optimized SLM process parameters compared to the values reported by different literature studies [14,31,32,33]. In order to evaluate the internal porosity inside the as-built cube samples, their outer sides were polished before the relative density was re-measured. As shown in Figure 5b, the higher relative density obtained from the polished samples reached 99.9% at an energy density of 50 J/mm^3^ (sample AS3), with a 0.1–1% reduction in porosity. By comparing the relative density between the as-built and the polished samples, it can be concluded that an increase in hatch spacing or scan speed parameters significantly increased the porosity on the sample surface, as illustrated in Figure 5c,d. This effect might result from the reduction of the material solidification rate at a higher scan speed and hatch spacing due to heat accumulation. The effect of the laser power indicated a significant impact of the increase of the melting rate and energy on the relative density of the as-built part. It is worth noting that the porosity percentage could be reduced after preheating the build platform prior to the sample build, as reported by Siddique et al. [34].

**Figure 6:**
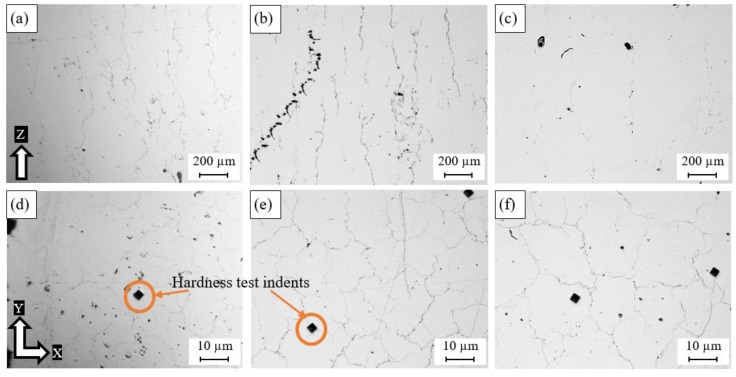
Pores observed inside the as-built Al6061 samples processed through different SLM parameters; (**a**,**d**) 8A, (**b**,**e**) 4A, and (**c**,**f**) 1A.

Figure 6 shows microscopic observations of the polished as-built Al6061 samples fabricated using the different SLM process parameters listed in Table 2. Different sizes of micro-cracks were observed between the samples along the Z-direction and the XY-plane. As shown in Figure 6, a lower porosity percentage was observed compared to the as-built AlSi10Mg samples. The keyhole pores were also reduced until they were hardly noticeable, with the exception of some spherical hydrogen pores. However, the relative density was relatively lower than that of the AlSi10Mg samples because of the presence of micro-cracks. The size of these micro-cracks depended on the thermal gradient between the deposited layer in addition to the CTE of the alloy and was also affected by the values of the SLM process parameters applied. Figure 6a–c shows the longitudinal microcracks formed along the Z-direction. Cracks with different sizes were obtained according to the applied SLM parameters. The smallest size and density of the cracks were observed after the applied energy density reached 102.8 J/mm^3^ in the 1A sample. However, no specific trend was detected between the energy density and the density of cracks, which is in agreement with Debroy et al. [35]. As illustrated in Figure 6d–f, the micro-cracks along the XY plane were shaped as semi-closed loops, similar in form to an equiaxed grain, but they were not entirely closed or sharp-edged. The results showed that the laser scan speed was the leading parameter affecting crack formation. The crack density, along with the building direction, increased along with the energy density from 40.5 to 76.9 J/mm^3^ at the same scan speed (1300 mm/s), as shown in Figure 6a,b respectively. However, the crack density displayed in Figure 6c was significantly reduced at a higher energy density (102.8 J/mm^3^) with a lower scan speed of 800 mm/s. Consequently, the scan speed had a more substantial effect on hot crack formation than the applied energy density, since it controlled the rate of solidification. The size of the semi-closed cracks formed in the XY plane tended to grow alongside the scan speed reduction, as noted in Figure 6d–f. The indents presented in Figure 6d–f were formed during the microhardness measurement that was reported by Maamoun et al. [25].

**Figure 7:**
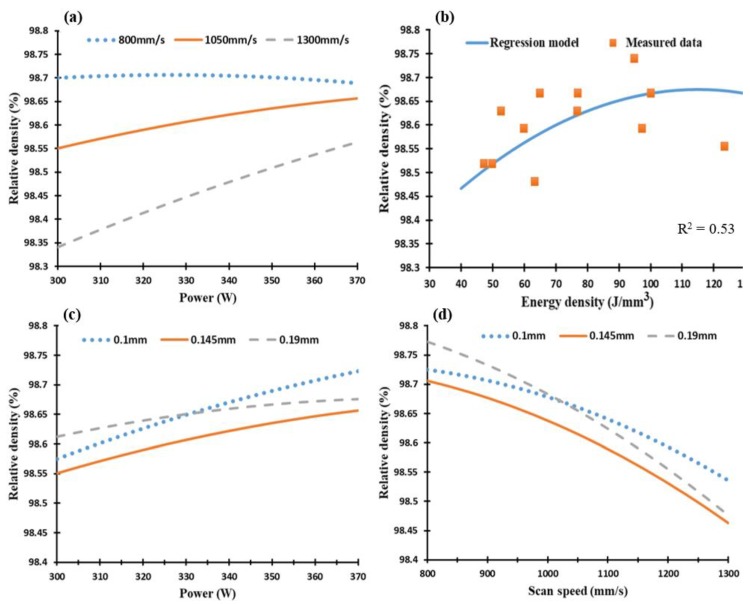
Relative density of the as-built Al6061 samples vs. (**a**) laser power (W), (**b**) energy density (J/mm^3^), (**c**) hatch spacing (mm), and (**d**) scan speed (mm/s).

Figure 7 presents the plots generated via the DOE analysis using the effect of the two combined process parameters on the relative density of the as-built part. It can be concluded that relative density tended to increase along with the laser power and energy density, while a lower rate of the laser scan speed led to denser parts. A significant relationship can be seen between laser power and scan speed, as illustrated in Figure 7c,d. A relative density average of 98.2 ± 0.5% was measured according to the selected process parameters, with the maximum relative density reaching 98.72% at energy density of 102.8 J/mm^3^ (sample 11A). These plots validated the trend obtained from the microscopic observations in Figure 6 and confirmed the effect of the laser scan speed on crack formation and relative density. The cracks observed inside the as-built parts could result from the hot crack phenomena which occur during material solidification owing to the combined chemical composition of the material. Kou et al. [36] reported that adding filler materials during welding to Al alloys susceptible to crack formation could eliminate the cracks and enhance the alloys’ weldability. This explains the crack-free structure obtained in the as-built AlSi10Mg parts, which had a high Si content compared to Al6061. 

### 3.3. Surface Topology

**Figure 8:**
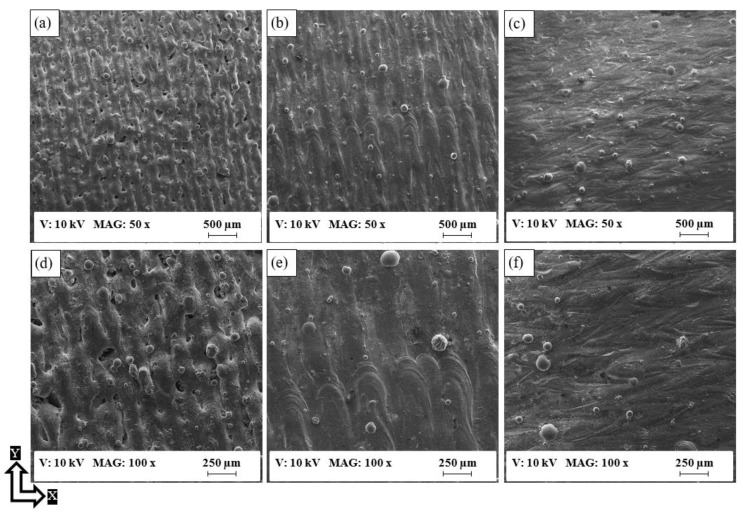
SEM observations of the as-built surface of AlSi10Mg samples; (**a**,**d**) AS8, (**b**,**e**) AS3, and (**c**,**f**) AS1.

The surface topology analysis of the as-built AlSi10Mg and Al6061 parts was conducted with SEM, displaying the 3D surface texture and mapping the relationship of the surface roughness with the SLM process parameters, according to the DOE analysis regression model. The surface defects of the as-built AlSi10Mg parts are exhibited in Figure 8 for different samples alongside the energy density increase. Figure 8a,d shows the rough surface obtained from the AS8 sample fabricated using a low energy density of 27 J/mm^3^. According to SEM observations, this high roughness resulted from surface pores forming because of a lack of fusion and of partially melted powder adhering to the surface. As shown in Figure 8b,e, an increase of energy density in the AS3 sample to 49.9 J/mm^3^, improved the surface roughness by eliminating noticeable surface pores and by reducing the density of the partially melted powder attached to the surface. However, the tracks of laser scanning were still visible with the commencement of balling phenomena. Figure 8c,f shows a better surface on the AS1 sample after applying a higher energy density of 63 J/mm^3^. This eliminated the tracks of laser scanning, but the balling effect was still present. The balling phenomena occur at higher energy density levels as a consequence of the surface tension generated around the melted powder particles. This represents an obstacle to the wetting of the underlying substrate layer by the melted powder [35]. It is also worthwhile to note that the effect of the balling phenomena increased as the energy density exceeded 65 J/mm^3^. As a result, the part build failed because of the detachment of the powder layer which had melted on the top of the underlying layer. 

**Figure 9:**
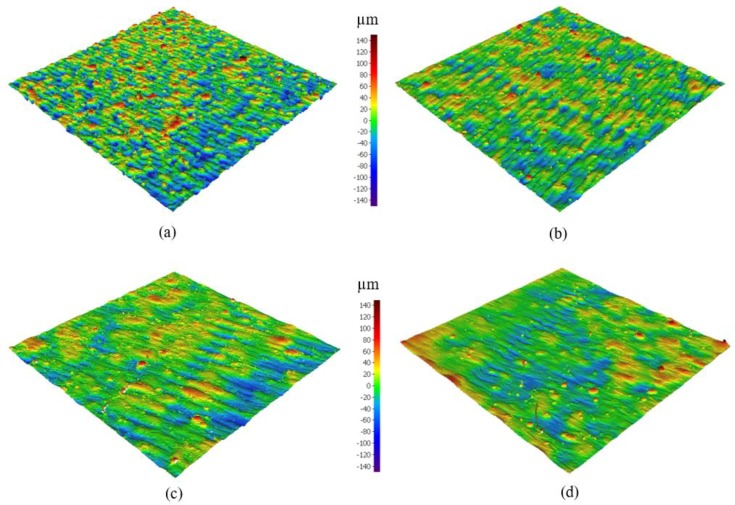
The 3D surface texture of the as-built AlSi10Mg samples; (**a**) AS8, (**b**) AS6, (**c**) AS3, and (**d**) AS1.

Figure 9 exhibits the 3D surface texture of the as-built AlSi10Mg samples; the results showed a significant improvement of the surface roughness alongside the increase of the energy density up to a specific limit. As shown in Figure 9a, applying a low energy density of 27 J/mm^3^ resulted in a rough texture with an average of 15 µm surface roughness. As illustrated in Figure 9b, the surface roughness decreased to 10 µm at a relatively high energy density of 40.5 J/mm^3^. The surface roughness continued to decrease until reaching the lowest value of 4.5 µm at an energy density of 65 J/mm^3^, as presented in Figure 9c,d.

**Figure 10:**
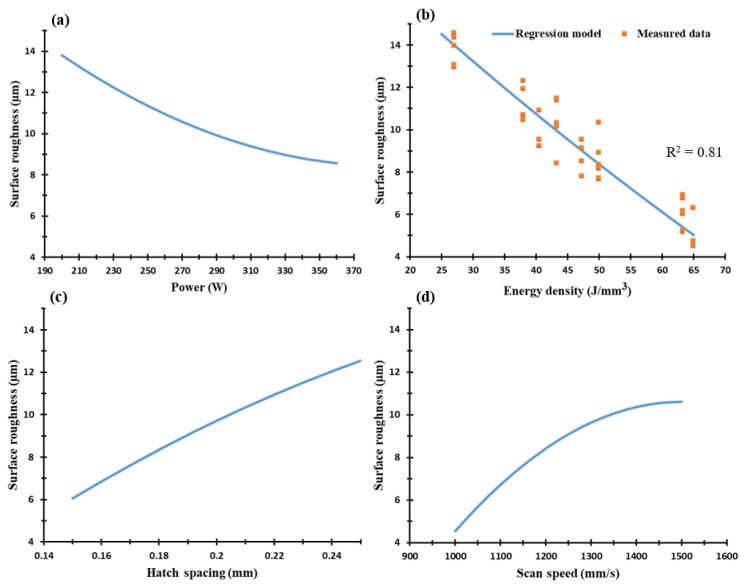
Surface roughness of the as-built AlSi10Mg samples vs. (**a**) laser power (W), (**b**) energy density (J/mm^3^), (**c**) hatch spacing (mm), and (**d**) scan speed (mm/s).

The mapping of the SLM process parameter effect on the surface roughness of the as-built AlSi10Mg parts is illustrated in Figure 10. The regression model generated by the energy density effect on the surface roughness showed a good agreement with the measured values. The laser power effect revealed the same trend as the energy density influence on the samples’ surface roughness, as shown in Figure 10a,b. The map displayed in Figure 10c also shows that the increasing of the hatch spacing value resulted in a more rough surface due to the decreasing overlap between the melted tracks, which agrees with the trend presented by Foster et al. [37]. The surface roughness also increased with the laser scan speed because of the reduction of the molten layer solidification rate, as presented in Figure 10d. A superior surface roughness of 4.5 µm was achieved with an E_d_ of 65 J/mm^3^ at 370 W laser power, 1000 mm/s scan speed, and 0.19 mm hatch spacing, which is in good agreement with the regression model. 

**Figure 11:**
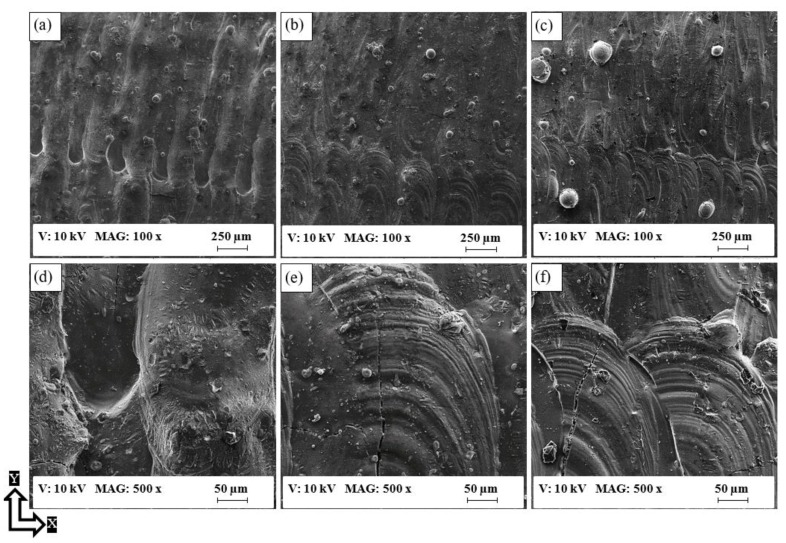
SEM observations of the as-built surface of Al6061 samples; (**a**,**d**) 7A, (**b**,**e**) 14A, and (**c**,**f**) 1A.

Figure 11 shows that the surface defects of as-built Al6061 parts were more significant than those of the AlSi10Mg parts. These defects were present in the partially melted powder adhering to the surface at a low energy density, surface porosity, and course shape of solidified tracks of laser scanning, as illustrated in Figure 11a. The surface finish gradually improved as the energy density increased from 50 to 123.3 J/mm^3^, as illustrated in Figure 11a–c. In Figure 11d–f, micro-cracks were also observed at a high microscopic magnification within a size of 50–200 µm, concentrated at the end of the laser tracks along the XY plane as a result of high thermal stress. These cracks adversely affected the surface roughness of the as-built Al6061 parts, which is why the SLM process parameters need to be optimized to reduce micro-crack formation. 

**Figure 12:**
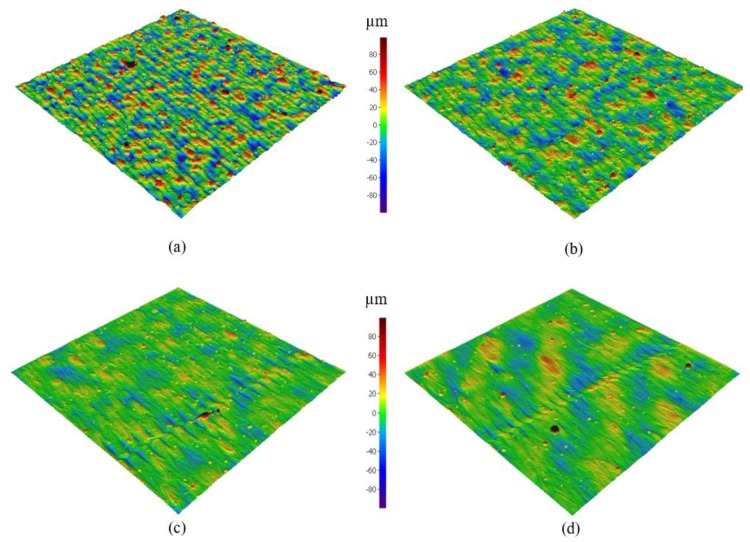
3D surface texture of the as-built Al6061 samples; (**a**) 8A, (**b**) 6A, (**c**) 14A, and (**d**) 11A.

The 3D surface texture of the Al6061 samples in Figure 12 confirms the trend of surface finish improvement with the application of a higher energy density. The energy density range of Al6061 (40.5–123.3 J/mm^3^) was shifted to a higher value compared to the limited E_d_ range of the AlSi10Mg alloy (27–65 J/mm^3^). This was due to the higher reflectivity and CTE of Al6061 compared to AlSi10Mg, which required more energy to completely melt the powder layer. However, the balling phenomena effect propagated at higher energy densities, limiting the applicable values of E_d_. 

**Figure 13:**
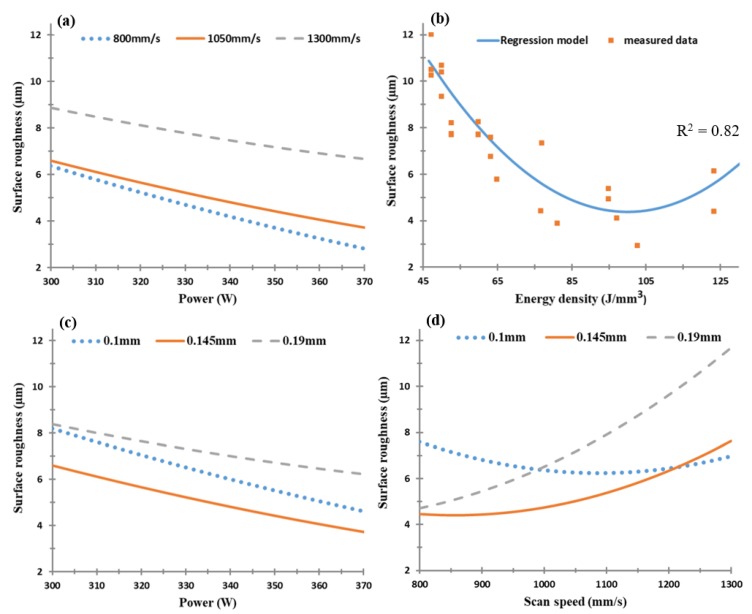
Surface roughness of the as-built Al6061 samples vs. (**a**) laser power (W), (**b**) energy density (J/mm^3^), (**c**) hatch spacing (mm), and (**d**) scan speed (mm/s).

The regression model derived from the surface roughness values versus the SLM process parameters is presented in Figure 13. The plots illustrate that the higher the laser power, the lower the roughness of the sample surface. The lowest surface roughness of 3 µm was obtained at 370 W laser power, 800 mm/s of scan speed, and 0.15 mm hatch spacing, which is in good agreement with the surface roughness measured for parts fabricated at an energy density of 102.8 J/mm^3^. In addition, no relationship was detected between the effect of the laser power on the surface roughness and the change in both scan speed and hatch spacing parameters, as illustrated in Figure 13b,c. However, a substantial relationship was noted between the scan speed and the hatch spacing effect on the surface roughness at a constant laser power value. The parabolic shape of the energy density impact indicated an optimum value of 102.8 J/mm^3^, which resulted in a better surface finish, as shown in Figure 13b.

### 3.4. Dimensional Accuracy

**Figure 14:**
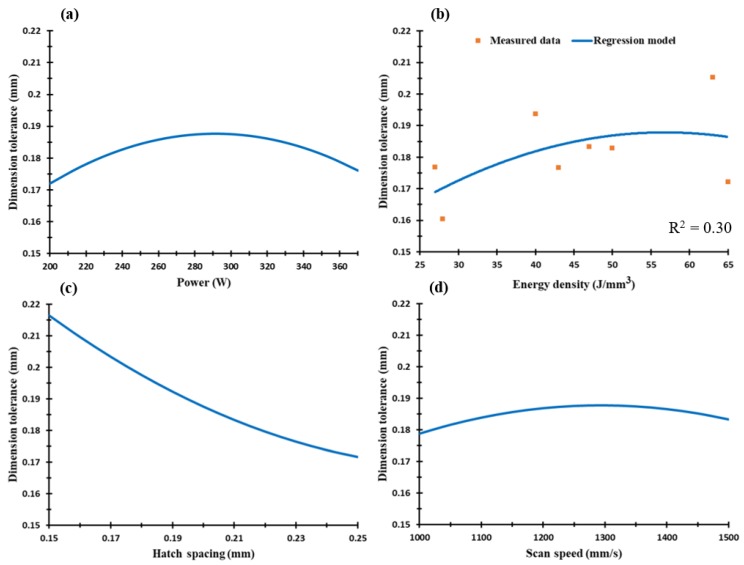
Dimension tolerance of the as-built AlSi10Mg samples vs. (**a**) laser power (W), (**b**) energy density (J/mm^3^), (**c**) hatch spacing (mm), and (**d**) scan speed (mm/s).

**Figure 15:**
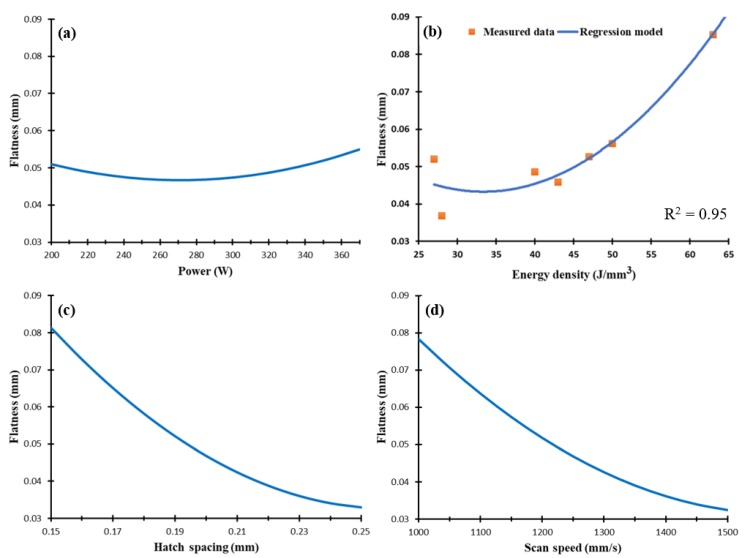
Surface flatness of the as-built AlSi10Mg samples vs. (**a**) laser power (W), (**b**) energy density (J/mm^3^), (**c**) hatch spacing (mm), and (**d**) scan speed (mm/s).

The dimensional accuracy analysis was performed according to the CMM measurements for both dimensional length tolerance and top surface flatness of the as-built AlSi10Mg and Al6061 parts. The values measured along the XY plane were used to generate the regression models which represented the effect of the SLM process parameters on each characteristic. Figure 14 shows the dimension tolerance of the average cube length for each sample. According to the recorded results, oversize dimension measurements were compared to the designed values, and there was no contraction observed in the cube sample length. The oversize in the cube length resulted from the balling effect and partial melted particles on the sample surface, which thus affected the outer surface stair-step profile [18]. After excluding the 0.02 mm laser beam offset, the dimension tolerance ranged from 0.15 up to 0.195 mm. Figure 14c shows that hatch spacing was the leading parameter affecting the dimension tolerance accuracy in addition to the laser power. The surface flatness difference between the samples tested showed a smaller range of change from 0.035 to 0.09 mm, due to the application of a small 30 µm layer thickness. Figure 15 shows the surface flatness behavior according to the change of the SLM process parameters. It was observed that the hatch spacing and scan speed were the main parameters affecting the parts’ surface flatness. The higher the scan speed and hatch spacing, the lower the surface flatness error obtained. Figure 15 also shows a good agreement between the regression model of the energy density effect on the surface flatness and the measured values. The results indicated that the surface flatness error increased together with an increase of the energy density. 

**Figure 16:**
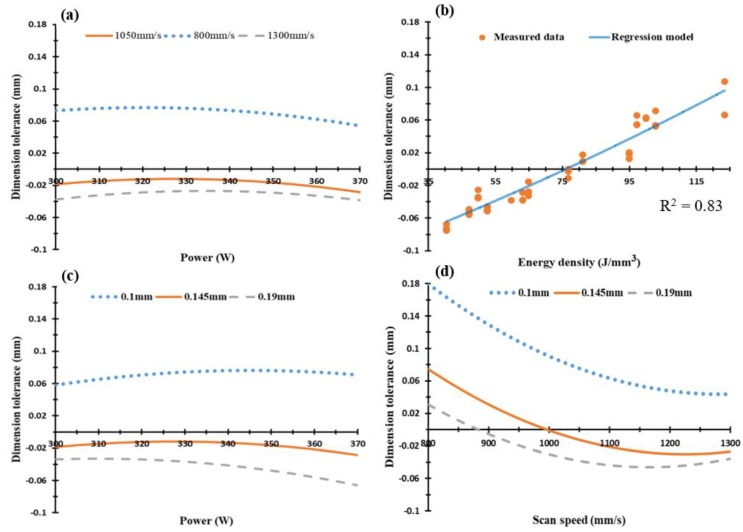
Dimension tolerance of the as-built Al6061 samples vs. (**a**) laser power (W), (**b**) energy density (J/mm^3^), (**c**) hatch spacing (mm), and (**d**) scan speed (mm/s).

The as-built Al6061 parts showed a different behavior for the dimensional tolerance values compared to the AlSi10Mg parts. As illustrated in Figure 16, the SLM parameters can affect the dimension tolerance by either expanding or contracting the dimensions, which could thus depend on the applied energy density. This might be caused by a change in the melt pool dimensions generated by the energy density [18]. The sample dimension tolerance showed a good agreement with the regression model curve. Figure 16b shows that an energy density higher than 76.8 J/mm^3^ resulted in higher dimension tolerance than the original energy density. However, an energy density below this level could lead to part dimension contraction due to the high CTE of Al6061, which resulted in an increased rate of heat dissipation and solidification. It is also noticed that part contraction occurred at lower rates of hatch spacing and at higher scan speeds.

**Figure 17:**
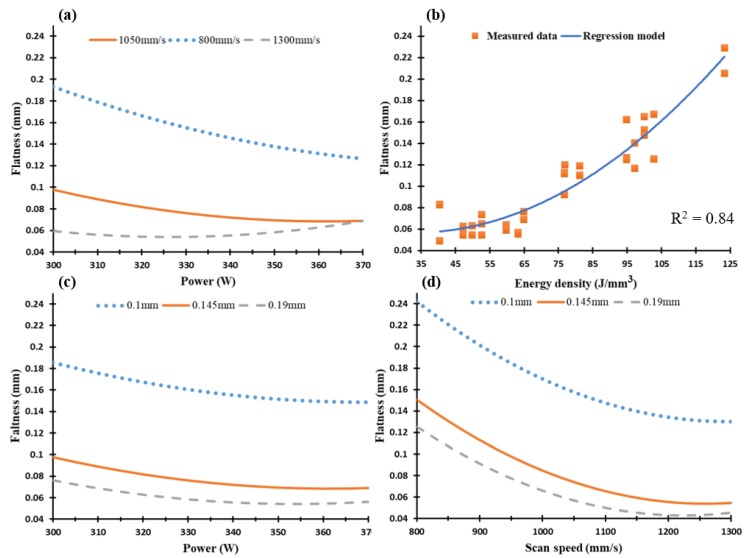
Surface flatness of the as-built Al6061 samples vs. (**a**) laser power (W), (**b**) eenergy density (J/mm^3^), (**c**) hatch spacing (mm), and (**d**) scan speed (mm/s).

In Figure 17, the surface flatness of the Al6061 samples was in the range of 0.05–0.24 mm, which was significantly higher than in the AlSi10Mg sample. This elevated surface flatness disparity might be due to the higher CTE of the Al6061 material which reduces heat accumulation inside the part. This difference in the surface flatness might also result from hot cracks forming inside the part after solidification, low Si content in Al6061, and the high reflectivity of Al6061. 

**Figure 18:**
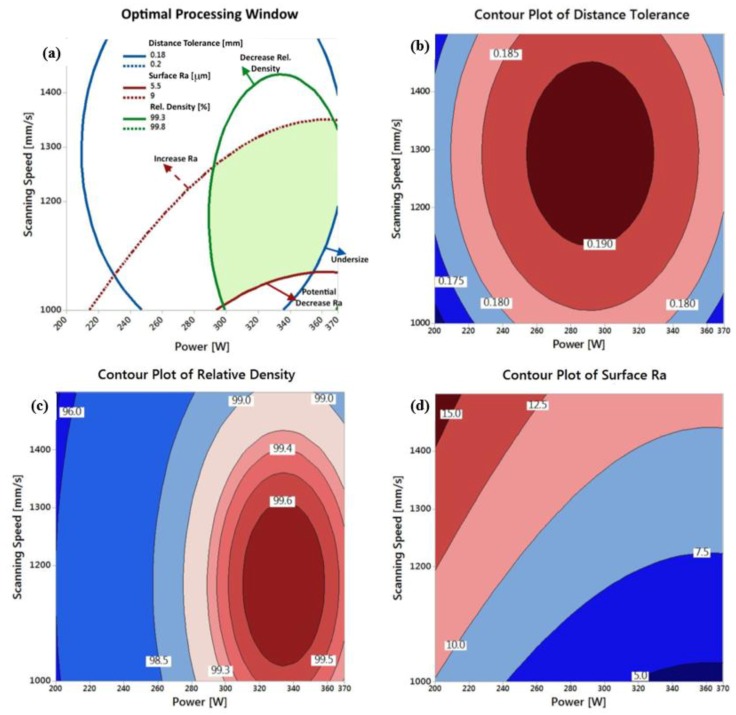
(**a**) Optimal processing window generated for the AlSi10Mg alloy at the hatch spacing value of 0.19 mm and the effect of laser power (W) and scan speed (mm/s) on (**b**) distance tolerance (mm), (**c**) relative density (%), and (**d**) surface roughness Ra (μm).

**Figure 19:**
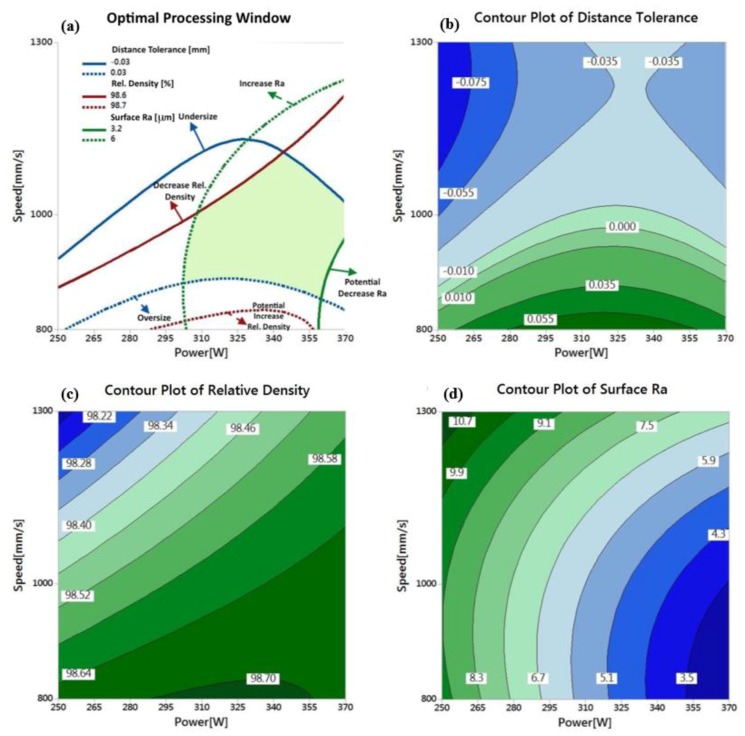
(**a**) Optimal processing window generated for the Al6061 alloy at the hatch spacing value of 0.15 mm and the effect of laser power (W) and scan speed (mm/s) on (**b**) distance tolerance (mm), (**c**) relative density (%) and (**d**) surface roughness Ra (μm).

A combination of the optimized range for each performance characteristic is presented in the process parameter map of the scan speed and laser power at a constant hatch spacing of 0.19 mm, as illustrated in Figure 18 and Figure 19. The process map for the as-built AlSi10Mg parts is displayed in Figure 18. This map presents an optimized range for the SLM process parameters to satisfy a surface roughness range from 5.5 to 9 µm, a relative density between 99.3% and 99.8%, and a range of dimensional tolerance from +0.18 to +0.2 mm. 

Figure 19 illustrates the process map for the as-built Al6061 parts that displays the optimized range for the scan speed and the laser power. The optimized process window shows a surface roughness improvement from 3.2 to 6 µm compared to the values obtained from the AlSi10Mg part process map. The dimensional tolerance is also optimized within a smaller range from −0.03 to +0.03 mm, with minimum reduction of dimensions compared to the severe contraction in Figure 16, which is avoided within the optimized process parameter range. However, the relative density of the optimized range has lower values that vary between 98.6% and 98.7%. 

## 4. Summary and Conclusions

The current study represents a comprehensive work that investigates the effect of SLM process parameters on the relative density, surface roughness, and dimensional accuracy of as-built AlSi10Mg and Al6061 parts [38]. A full characterization of both materials’ powders was presented. DOE was used to investigate relative density, porosity, surface roughness, surface defects, and dimensional accuracy. Regression models and trends were obtained from the measured data. The results showed the following different characteristic behaviors for each material:

1. Powder morphology revealed that AlSi10Mg and Al6061 possess a spherical particle shape interspersed with of elongated particles in a considerable percentage. PSD showed a positively skewed distribution within a range of 12–120 µm.
2. The rate of energy density affected the relative density and porosity formation inside the as-built parts. The optimum range of energy density was 50–60 J/mm^3^, which resulted in relative density reaching 99.7%. The relative density of the polished samples reached 99.9%, with a 0.1% internal porosity. The higher rates of energy densities contributed to the formation of large hydrogen spherical pores, while the lower rates resulted in keyhole pores because of the lack of powder fusion.
3. For the Al6061, the maximum relative density measured was 98.72% using an energy density of 102.8 J/mm^3^ and an 800 mm/s scan speed. A relationship between the scan speed and the laser power was noted, whereby the highest relative density was achieved at a low scan speed and high laser power. The relative density of the Al6061 parts showed lower values compared to the relative density of AlSi10Mg, due to lower Si content, which increased the CTE and caused the formation of hot cracks inside the as-built Al6061 parts.
4. The surface topology was significantly affected by the energy density applied to both materials. The surface roughness reduced alongside the increase of energy density. For the AlSi10Mg samples, the minimum surface roughness was 4.5 µm at 65 J/mm^3^. For the Al6061 parts, an energy density of 102.8 J/mm^3^ resulted in the best surface roughness of 3 µm. The energy density was limited to a maximum of 65 J/mm^3^ for AlSi10Mg and to 123.3 J/mm^3^ for Al6061, to avoid delamination and failure of part building.
5. For AlSi10Mg, the dimensional tolerance varied between an oversize of 0.15 and 0.195 mm. The best surface flatness could be obtained with higher hatch spacing and scan speeds.
6. For the Al6061 parts, the lowest dimensional tolerance was achieved using an energy density of 76.8 J/mm^3^. Contraction of the part dimension was observed at lower energy densities, and oversized part dimension was detected at higher energy densities. The surface flatness of Al6061 was superior to that of the AlSi10Mg parts.
7. An optimal processing window was developed for each material to illustrate the mutual connection between relative density, surface topology, and dimensional accuracy, with the goal of achieving a high-quality end product.

## Figures and Tables

**Table 1:** The selective laser melting (SLM) process parameters used for building the AlSi10Mg samples. P: laser beam power, V_s_: laser scan speed, D_h_: hatch spacing between scan passes, E_d_: energy density.

Sample #	P (W)	V_s_ (mm/s)	D_h_ (mm)	E_d_ (J/mm^3^)
**AS1**	370	1000	0.19	65
**AS2**	370	1300	0.15	63.2
**AS3**	370	1300	0.19	50
**AS4**	350	1300	0.19	47.2
**AS5**	370	1500	0.19	43.3
**AS6**	300	1300	0.19	40.5
**AS7**	370	1300	0.25	38
**AS8**	200	1300	0.19	27

**Table 2:** The SLM process parameters applied for fabricating the Al6061 samples.

Sample #	P (W)	V_s_ (mm/s)	D_h_ (mm)	E_d_ (J/mm^3^)	Sample #	P (W)	V_s_ (mm/s)	D_h_ (mm)	E_d_ (J/mm^3^)
**1A**	370	1000	0.1	123.3	**11A**	370	800	0.15	102.8
**2A**	300	1000	0.1	100	**12A**	350	800	0.15	97.2
**3A**	370	1300	0.1	95	**13A**	370	800	0.19	81.1
**4A**	300	1300	0.1	76.9	**14A**	350	800	0.19	76.8
**5A**	370	1000	0.19	65	**15A**	370	1300	0.15	63.2
**6A**	300	1000	0.19	52.6	**16A**	350	1300	0.15	59.8
**7A**	370	1300	0.19	50	**17A**	370	1300	0.19	50
**8A**	300	1300	0.19	40.5	**18A**	350	1300	0.19	47.2

**Table 3:** Energy X-ray dispersive spectroscopy (EDS) analysis of the Al6061 and AlSi10Mg powders’ chemical composition.

Element	Si	Mg	Cu	Fe	Al
**Al6061 wt %**	1.2	0.77	0.32	0.90	Balance
**AlSi10Mg wt %**	11.34	0.28	0.08	0.32	Balance

**Table 4:** Values measured for the particle size distribution of the Al6061 and AlSi10Mg powders.

Sample Type		D (0.1)	D (0.5)	D (0.9)
Al6061 Powder	Diameter (μm)	22.83	41.27	71.92
AlSi10Mg Powder		23.16	39.62	66.55
